# The Influence of Risk Factor Modification on Atrial Fibrillation Outcomes and Their Impact on the Success of Catheter Ablation

**DOI:** 10.31083/RCM27175

**Published:** 2025-03-21

**Authors:** Nakul Chandan, Vishnu Ashok, Taesoon Hwang, Ven Gee Lim, Thomas Lachlan, Helen Eftekhari, Gordon McGregor, Faizel Osman

**Affiliations:** ^1^Institute for Cardiometabolic Medicine, University Hospitals Coventry & Warwickshire NHS Trust, CV2 2DX Coventry, UK; ^2^Centre for Healthcare & Communities, Coventry University, CV1 5FB Coventry, UK; ^3^Warwick Medical School, University of Warwick, CV4 7HL Coventry, UK

**Keywords:** atrial fibrillation, risk factor modification, cardiac rehabilitation, AF ablation outcome

## Abstract

The global prevalence of atrial fibrillation (AF) is growing with a significant increase in AF burden. The pathophysiology of AF is complex and exhibits a strong relationship with modifiable lifestyle AF risk factors, such as physical inactivity, smoking, obesity, and alcohol consumption, as well as co-morbidities, such as hypertension, diabetes mellitus, and cardiovascular disease. Current evidence suggests that cardiac rehabilitation and lifestyle risk factor modification can potentially lower the overall AF burden. Additionally, AF ablation can be an effective treatment for a rhythm control strategy, but reducing AF recurrences post-catheter ablation is paramount. Thus, addressing these modifiable lifestyle risk factors and co-morbidities is critical, as the recent 2024 European Society of Cardiology AF guidance update highlights. A comprehensive approach to treating these risk factors is essential, especially given the rising prevalence of AF. This article provides a state-of-the-art update on the evidence of addressing AF-related risk factors and co-morbidities, particularly in patients undergoing AF ablation.

## 1. Introduction

Atrial fibrillation (AF) is the most common cardiac arrhythmia. Further, AF represents an ever-growing global epidemic, with estimates demonstrating that more than 59 million individuals lived with AF globally in 2019, and the prevalence has doubled over the previous two decades [1, 2]. This trend was confirmed in international data, demonstrating a clear rise in costs and burden on healthcare systems because of increased morbidity and all-cause mortality, largely driven by thromboembolic events (i.e., stroke, myocardial infarction), cognitive decline, and heart failure (HF) [2, 3, 4]. Subsequently, this trend has been partially explained by advances in detection modes and improved survival but also due to the increased accumulation of co-morbidities that comprise AF risk factors [3]. While the pathogenesis of AF has a genetic component, environmental and lifestyle risk factors and co-existing medical and cardiovascular (CV) conditions play a major role by contributing to the electrical, structural, and functional remodeling of the heart [5, 6]. Suboptimal management of these risk factors can lead to increased arrhythmia burden, disease progression, and incidence of adverse events associated with AF [7].

**Fig. 1.**
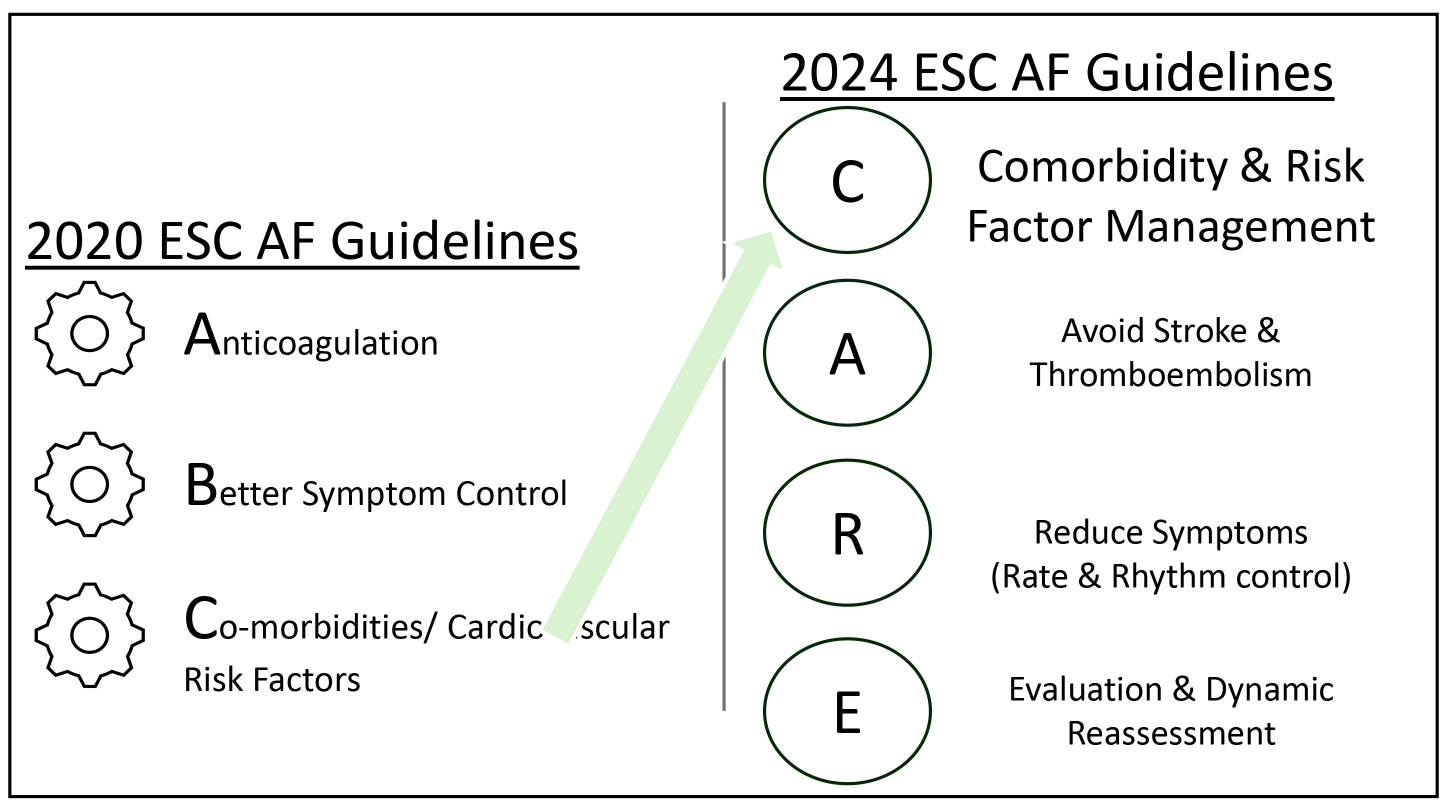
**The change in approach to atrial fibrillation management, as demonstrated in the 2024 ESC AF guideline update with prioritization of co-morbidities and AF risk factor management (adapted from the ESC 2024 Guidelines [7])**. AF, atrial fibrillation; ESC, European Society of Cardiology.

Historically, AF management primarily emphasized thromboembolic risk reduction and HF prevention through rate and rhythm control strategies [8, 9]. However, there has been a recent paradigm shift, with current international guidelines prioritizing the management of co-morbidities and risk factor modification to optimize patient outcomes [7, 10]. Indeed, the recent 2024 European Society of Cardiology (ESC) updated AF guidelines [7] advocate for the Co-morbidity, Avoid stroke, Reduce symptoms, Evaluation (CARE) approach (Fig. 1, Ref. [7]), placing increasing emphasis on the careful search for co-morbidities and risk factors and applying the strategy in all patients diagnosed with AF as a priority. Middeldorp *et al*. [11] have shown that the progression of AF from occasional episodes to persistent, long-standing persistent, and ultimately permanent forms can be halted and reversed by addressing these underlying risk factors. Despite the growing evidence supporting the critical role of risk factor modification, there is also evidence that these risk factors are often overlooked as presented in real-world data [12].

**Fig. 2.**
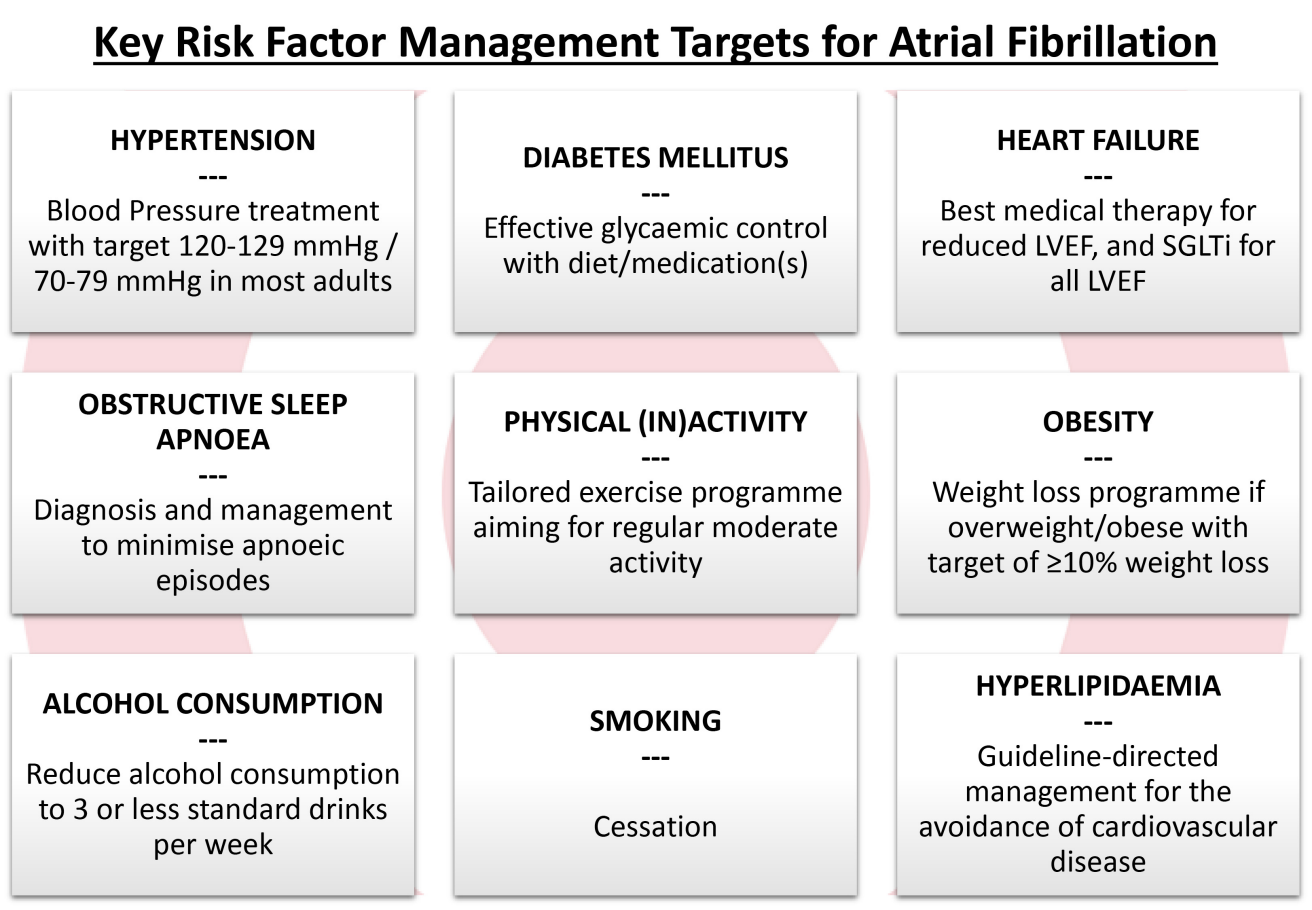
**Key risk factors for atrial fibrillation and their management targets (adapted from ESC 2024 AF Guidelines [7])**. LVEF, left ventricular ejection fraction; SGLTi, sodium-glucose co-transporter 2 inhibitor; AF, atrial fibrillation; ESC, European Society of Cardiology.

This review article aims to provide an update on the current evidence regarding the impact of risk factor management on outcomes, especially following AF ablation, in light of the renewed emphasis on AF risk factor modification, as highlighted by the 2024 International Consensus Statement and 2024 ESC AF guidelines [7, 13] (see Fig. 2, Ref. [7]).

## 2. AF Management, Rhythm Control, and AF Ablation

Over the last two decades, landmark studies have emphasized AF management through symptom reduction; however, the rationale for long-term rhythm control therapy has recently evolved [7]. Studies had initially found no mortality benefit and potentially increased hospitalization with pharmacological rhythm control versus rate-control [14, 15]; conversely, multiple studies demonstrated the positive impact of rhythm control strategies on quality of life when sinus rhythm was maintained [16, 17, 18]. In 2020, the EAST-AFNET 4 trial [19] demonstrated the benefit of an early rhythm control strategy in patients whose AF was ≤1 year compared to usual care that focused on rhythm control only for managing AF-related symptoms. In the early rhythm control arm, 86.8% of those who were initially managed using anti-arrhythmic drugs (AAD) were associated with a lower primary composite outcome of CV death, stroke, or hospitalization with worsening of HF or acute coronary syndrome. Nonetheless, the effective use of all AAD for rhythm control is constantly plagued by the risk of serious cardiac and extracardiac adverse effects [7]. Notably, drug safety often takes precedence over efficacy in determining their use.

Over the last 30 years, evidence for catheter ablation of AF as a viable first-line option for AF management has evolved [20, 21] and has demonstrated benefits with or without AAD [22, 23, 24, 25, 26, 27, 28, 29]. In the EAST-AFNET 4 trial, at a 2-year follow-up, 19% of the early rhythm control arm had received catheter ablation [19]. In the CABANA trial [16], which compared catheter ablation with AAD in the treatment of symptomatic AF, the authors demonstrated no significant difference in the primary composite outcome of death, disabling stroke, serious bleeding, or cardiac arrest over a median 4-year follow-up. However, when interpreting the results of this study, one must consider the estimated treatment effect, which was affected by lower-than-expected event rates and treatment crossovers, with 27.5% of the drug therapy arm ultimately receiving catheter AF ablation. Furthermore, secondary outcomes (death or CV hospitalization and AF recurrence) significantly favored catheter ablation. A recent review [30] found catheter AF ablation as a first-line therapy for AF was associated with significant improvements in arrhythmia-related outcomes, symptoms, quality of life, and lower rates of adverse events. Primarily designed as an adjunct to cardiac surgical patients to reduce stroke risk in atrial fibrillation patients, left atrial appendage (LAA) ligation was shown to modify the atrial structural and electrical properties influencing AF dynamics [31, 32]. Subsequently, the role of the LAA has been explored as an arrhythmogenic focus, indicating that LAA ligation can potentially reduce AF triggers but may also create new substrates in some individuals [31, 32, 33, 34]. With the increasing accessibility to percutaneous methods for LAA ligation, the recent aMAZE trial, comparing adjunctive LAA ligation with pulmonary vein isolation (PVI) to PVI alone, met safety and closure efficacy, but did not meet prespecified efficacy for freedom from atrial arrhythmias at 12 months [35]. Catheter ablation, similar to many cardiac interventional procedures, is not without risk and carries a variable success rate of 40–80% for a first-time procedure, depending on AF type and an individual’s other clinical factors [36, 37]; paroxysmal AF carries a higher success rate (70–80%) compared with persistent and long-standing persistent AF (40–60%). AF catheter ablation also has demonstrable attrition at follow-up, with estimated AF recurrence rates of single-procedures reaching up to 30–70% at longer-term follow-ups [36, 38], with repeat ablations often required to achieve higher success rates [36, 37, 38, 39]. This has been especially noted in those with persistent AF and those with multiple co-morbidities and attributable risk factors for AF.

With healthcare systems evaluating treatments for sustainability, including cost-effectiveness, lower success rates with known attrition over time can significantly impact decisions and willingness to pay for expensive therapies, raising concerns about exactly who and how many patients can undergo an AF ablation procedure. Catheter ablation can be a cost-effective treatment strategy for patients with AF, particularly those with early-onset AF, as it may delay progression to more advanced forms of the condition. Therefore, greater utilization of ablation, especially in earlier stages of disease, can potentially deliver both clinical and economic benefits [40].

Atrial cardiomyopathy is widely recognized as a substrate for arrhythmic recurrences when aiming for rhythm control; however, evidence suggests that catheter ablation alone may not address this progressive atrial substrate remodeling [41]. Moreover, targeting additional atrial substrate during the initial AF ablation offers no advantage over PVI alone, suggesting its limited effect on post-ablation atrial remodeling [7]. Conversely, emerging evidence demonstrates the impact of AF risk factors on the progression and potential reversibility of the underlying atrial substrate. The following sections discuss the roles of various AF risk factors and their influence on the pathogenesis, incidence, and recurrence of AF, particularly in AF ablation.

## 3. Risk Factors for Atrial Fibrillation 

### 3.1 Hypertension

The Framingham Heart study estimated that the presence of hypertension increased the likelihood of developing AF by 40–50% [42]. In an ovine model, hypertension has been associated with early and progressive changes in atrial remodeling [43, 44]. Indeed, left ventricular hypertrophy and stiffening, reduced diastolic filling and increased left atrial (LA) volumes because of the increased afterload with hypertension; LA dilatation, reduced LA function, adverse electrophysiological changes, and increased interstitial inflammation and fibrosis, which all serve as substrates for AF.

The blood pressure (BP) control target suggests strict control, avoiding hypertensive ranges [45]. A dose-response relationship has been demonstrated, showing a greater reduction in the risk of new AF with lower systolic BP (SBP): a SBP 131–141 mmHg and SBP ≤130 mmHg derived a 24% and 40% lower risk, respectively [46]. More recently, a large cohort study exploring ideal BP in AF patients demonstrated BP >130/80 mmHg and BP <120/80 mmHg were associated with a higher risk of major adverse CV events, suggesting an optimal target of 120 to 129/<80 mmHg [47]. The 2024 ESC AF guidelines [7] confirm a target SBP of 120–129 mmHg and diastolic BP (DBP) of 70–79 mmHg, where tolerated, with SBP <140 mmHg as acceptable in frailer patients. Some evidence suggests anti-hypertensive type matters, with evidence for renin–angiotensin system (RAS) inhibitors versus others, despite similar BP controls [48, 49, 50, 51]. These RAS inhibitors have shown greater potential to reduce the incidence of new-onset AF, greater efficacy in preventing AF recurrence in mild hypertension, and significantly reduce progression to persistent AF. This effect is likely due to RAS inhibitors having demonstrated beneficial effects beyond the control of hypertension on cardiac electrical and structural remodeling, particularly LA fibrosis and dilatation [52].

When assessing outcomes of AF ablation, multiple studies have shown hypertension, and especially uncontrolled hypertension, was independently associated with and a predictor for AF recurrence [53, 54, 55, 56, 57]. Zylla *et al*. [58] explored long-term outcomes of the German Ablation Registry of 626 patients; patients with hypertension were older, had more CV co-morbidities, and presented with more persistent forms of AF. Though the study found no statistical difference in AF recurrence rates, freedom from AAD, and requirement for repeat ablation, hypertensive patients had higher hospitalization rates and complained of more dyspnea and angina. Similar to the optimal range for blood pressure in patients with AF, the SMAC-AF study [59] demonstrated aggressive BP (target <120/80 mmHg) versus standard BP (target <140/90 mmHg) control before AF ablation, did not reduce atrial arrhythmia recurrence, but instead lead to a higher rate of hypotension requiring medication adjustments.

Randomized clinical trials have investigated the effects of combining renal denervation (RDN) and PVI in patients with AF and hypertension. Pokushalov *et al*. [60] considered patients with refractory symptomatic AF and resistant hypertension, demonstrating that the addition of RDN to PVI resulted in significantly higher rates of freedom from AF recurrence at 12 months, alongside notable reductions in blood pressure, compared to PVI alone. Similarly, the ERADICATE-AF trial [61], which focused on patients with paroxysmal AF and sub-optimally controlled hypertension, showed that combining RDN with PVI led to a significant reduction in AF recurrence, AF burden, and blood pressure levels relative to PVI alone. These findings suggest that RDN, when performed in conjunction with catheter ablation, may offer enhanced rhythm control and improved clinical outcomes in hypertensive AF patients, likely through modulation of sympathetic nervous system activity. Further large-scale studies are warranted to confirm these results and establish the long-term efficacy and safety of this combined approach. Furthermore, a lack of sham procedure controls for the RDN components in these studies represents a criticism, as this feature affected outcomes of renal denervation trials solely focused on hypertension management.

### 3.2 Diabetes Mellitus

Individuals with diabetes mellitus (DM) have a significantly higher likelihood of developing AF, with a meta-analysis of 31 studies, including more than 10 million participants, demonstrating a dose-response relationship between incremental blood glucose and the risk of AF [62]. Estimates concluded a 20% increase in the pre-diabetic range and 28% with diagnosed DM. The ARIC study [63] described each 1% rise in HbA1c drove a 13% higher risk of AF. A registry-based cohort study concluded that DM was associated with an overall 35% higher risk than age- and sex-matched controls during a 13-year follow-up [64]. This study also suggested excess risk for AF, where there was the presence of poor glycemic control and evidence of renal complications. Similarly, the Framingham Heart Study [65] demonstrated an associated risk of DM with AF, as high as 40% in men and 60% in women. The risk from DM is likely multi-faceted, with mechanisms including mitochondrial dysfunction, oxidation, fibrosis, inflammatory fatty accumulation and infiltration, impairment of calcium transport, and thrombogenesis, leading to cardiac structural, electrical, and autonomic remodeling [66].

Arrhythmia-free survival after catheter ablation is known to be significantly lower among patients with DM, with risk associated particularly with poor glycemic control [67, 68]. However, a review of a large cohort from the German Ablation Registry demonstrated no increased atrial arrhythmia recurrence associated with DM [69]. A meta-analysis of 15 studies, including 1464 patients, also showed AF ablation safety and efficacy similar to the general population, particularly in a younger cohort with reasonable glycemic control. However, a higher frequency of redo-ablations in DM patients was required to achieve a similar efficacy [70]. One meta-regression analysis in this study demonstrated a higher baseline glycated haemoglobin (HbA1c), along with advanced age and higher body mass index (BMI), related to a higher incidence of arrhythmia recurrence.

Reinforcing the benefits of good glycemic control in DM patients due to undergoing AF ablation, a retrospective study of nearly 300 patients [71] demonstrated both avoidance of DM and increased glycemic control pre-ablation reduced arrhythmia recurrence post-ablation; HbA1c levels of >9% compared with <7%, showed a 68.75% versus 32.4% risk of recurrence post-AF ablation over a mean of 2 years, and those with a 10% reduction in HbA1c over the 12 months pre-ablation versus evidence for worsening glycemic control, demonstrated a striking difference of 2% and 91% risk of recurrence, respectively.

Recent studies have investigated the potential specific impact of sodium–glucose co-transporter-2 inhibitors (SGLTis) on AF recurrence following catheter ablation in patients with DM [72, 73]. A retrospective analysis has demonstrated that SGLTi use is associated with a significantly reduced risk of arrhythmia recurrence after AF ablation, thereby decreasing the requirement of subsequent interventions: cardioversion, AAD, or repeat ablation [72]. Similarly, a prospective study and a meta-analysis showed that SGLTi therapy correlates with improved maintenance of sinus rhythm post-ablation in DM patients [73]. These observed benefits are suggested to be attributable to the effects of SGLTis on weight reduction, blood pressure control, intravascular volume management, and mitigation of atrial fibrosis and adverse cardiac remodeling [72]. These findings highlight the potential role of SGLTis in enhancing outcomes following catheter ablation in patients with DM. However, further large-scale, prospective studies are needed to confirm these results and clarify the proposed underlying mechanisms.

### 3.3 Heart Failure

HF and AF often co-exist, frequently complicating one another [74, 75]. HF is a key determinant for the prognosis of AF patients, including the recurrence and progression of arrhythmia. The Framingham cohort demonstrated that more than half of those with new HF had associated AF, and 37% of those with new AF diagnoses had HF [75]. The risk of new AF in HF can be attributed to multiple mechanisms related to neurohormonal, structural, and ultrastructural changes, including atrial pressure overload and enlargement, altered myocardial conduction, maladaptive gene expression, and cardiac remodeling [74].

Owing to the co-existence of the two conditions and the rise in options for medical therapy for HF, multiple studies containing large proportions of participants with AF have demonstrated mortality benefits, reduced hospitalization, and reduced urgent HF visits [76, 77, 78, 79, 80]. A recent meta-analysis [81] showed that SGLTis might only reduce HF hospitalization and CV death to a similar degree in those with or without AF but went on to build an association between SGLTis with reduced total and serious AF event rates. Our recent review postulated that SGLTis may have anti-arrhythmic effects via action on cardiac autonomic function [82].

Multiple randomized trials have demonstrated the potential of AF ablation to improve clinical outcomes in patients with AF and symptomatic heart failure with reduced ejection fraction. The PABA-CHF trial [83] showed that PVI was superior to atrioventricular node ablation and biventricular pacing in improving functional capacity, quality of life, and left ventricular ejection fraction (LVEF). Similarly, the ARC-HF trial [84] found that catheter ablation led to significant improvements in exercise capacity, quality of life, and the neurohormonal states of patients compared to a pharmacological rate control strategy. Further reinforcing this notion, the AATAC trial [85] found that catheter ablation resulted in higher rates of freedom from AF, reduced mortality, fewer hospitalizations, and improvements in LVEF and exercise capacity compared to amiodarone therapy. Marrouche *et al*. [86] also demonstrated catheter AF ablation in HF patients intolerant or unwilling to take AADs was associated with a significantly lower rate of a composite endpoint of death from any cause or hospitalization for worsening HF when compared to medical therapy (rate or rhythm control). In a recent study, randomizing patients with AF and end-stage HF, the combination of catheter ablation and guideline-directed medical therapy was associated with a lower likelihood of a composite of death from any cause, implantation of a left ventricular assist device, or requirement for urgent heart transplantation, compared with medical therapy alone [87]. Together, these studies position catheter ablation as a potentially valuable therapeutic strategy in this patient population.

Given this evidence, optimal management of HF may benefit from increasing arrhythmia-free survival after rhythm control by modifying and reversing substrates for AF. There is, however, limited evidence for specific interventions. The RACE 3 trial [88], which combined best medical therapy with cardiac rehabilitation for patients with mild-to-moderate HF and persistent AF, increased maintenance of sinus rhythm after cardioversion at 12 months, but the results were no longer present at the 5-year follow-up.

### 3.4 Obstructive Sleep Apnea

In recent years, obstructive sleep apnea (OSA) has been increasingly recognized as an important risk factor for AF. OSA is characterized by apneic episodes resulting from pharyngeal collapse [7]. Meanwhile, increased vagal activation from repetitive transient hypoxemia has been proposed as a factor that may affect the atrial effective refractory period (ERP) and thereby increase susceptibility to the development of AF [89]. Furthermore, hemodynamic changes from long-standing OSA can cause an increase in LA pressure, leading to LA dilatation, which can lead to the development of AF [90]. Chronic OSA demonstrated in rat models induced cardiac remodeling, which is known to promote AF with conduction abnormalities related to connexin dysregulation and is associated with increased inflammatory and prothrombotic states and myocardial fibrosis [91].

Growing evidence suggests that outcomes post-AF ablation are poorer in patients with OSA, with meta-analytical data showing up to 70% higher risk of AF recurrence post-catheter ablation in this cohort [92]. In a study of 62 patients with OSA undergoing PVI for symptomatic AF, arrhythmia-free survival was better in those receiving continuous positive airway pressure (CPAP) than non-users [93], with other observational studies demonstrating similar results [94, 95]. We are currently undertaking the OSCA trial [96], which is a two-center nested cohort study using a Reveal LINQ II (Medtronic, Minneapolis, MN, USA) implantable loop recorder (ILR) to identify precise arrhythmia (atrial/ventricular) incidence in patients with moderate–severe OSA. We recruited 200 randomized patients 1:1 to standard care alone or standard care + ILR (+ Holter monitor at baseline and 12 months). The primary objective was to compare arrhythmia detection over 3 years between the two groups. Cardiac autonomic function was assessed in the ILR arm at baseline and 12 months post-CPAP. The secondary objectives explored the mechanisms linking OSA and arrhythmia using cardiac autonomic function parameters based on Holter recordings and circulating biomarkers (high sensitivity Troponin-T, N-terminal pro-B-type natriuretic peptide, matrix metalloproteinase-9, fibroblast growth factor 23, high sensitivity C-reactive protein, interleukin-6 and tumor necrosis factor-α) before and after CPAP initiation in the ILR-arm. The OSCA trial is due for report in late 2025.

### 3.5 Obesity

The relationship between obesity and AF is well established, with increasing evidence linking obesity as an independent risk factor for AF [97]. Results from the ARIC study [98] have shown that being overweight and obese BMI ≥25 kg/m^2^) accounted for about 18% of AF incidents, making obesity the second strongest AF risk factor. Obesity, as demonstrated in a cohort of AF patients, has been associated with increased rates of dilated cardiomyopathy, hypertension, DM, and OSA—all risk factors for AF in their own right [99]. Structural and electrical atrial remodeling have been implicated as the pathological changes secondary to long-standing obesity [100, 101]. Evidence for substrate progression is rooted in increased LA diameter, pressure, blood volume, central blood volume, systemic vascular resistance, epicardial and pericardial fat deposition, and conduction slowing [100, 101, 102, 103]. A previous meta-analysis of 51 studies demonstrated a 29% increase in AF incidence with an increase in BMI by 5 units and a 13% increase in AF recurrence post-AF ablation [104]. Comparable results have also been reported in other large studies, including the Women’s Health Study and the Framingham Heart Study [105, 106]. A retrospective review of 2715 patients, unsurprisingly, related BMI to the type of AF; it found a decreasing incidence of paroxysmal AF and increasingly persistent forms of AF with higher BMIs [99]. Equally, an increase in AF recurrence post-ablation with increased BMI (≥35 kg/m^2^) was also demonstrated, and importantly, safety risks increased alongside morbid obesity (BMI ≥40 kg/m^2^).

The distribution of body weight also appears to be significant. In a registry study involving patients with a BMI between 18.5 and 23 kg/m^2^, those with a waist circumference exceeding 80 cm in females and 90 cm in males had a higher risk of developing AF [107]. A prospective cohort database of 44,135 employees from a coal mining company identified similar waist circumference thresholds as independent predictors of AF incidence, even after adjusting for BMI [108]. These findings may explain why some studies have reported a linear relationship between BMI and AF risk, while others have observed a ‘j-shaped’ association.

Weight reduction has positively impacted cardiac structure, reduced AF events on ambulatory recording, and reduced AF symptom burden. The LEGACY-AF study [109] demonstrated long-term sustained weight loss, particularly with avoidance of weight fluctuation, was associated with a dose-dependent reduction in AF burden and maintenance of sinus rhythm. It is believed the benefit was derived from the coinciding favorable changes in cardiometabolic risk factor profile, inflammatory state, and improved cardiac remodeling. The ARREST-AF study [110] demonstrated increased post-ablation AF-free periods and greater weight loss with aggressive risk factor modification to address obesity. Studies on the effects of bariatric surgery on the outcomes of AF ablation, including a retrospective analysis of 239 patients with BMIs >35 kg/m^2^ and 40 kg/m^2^, showed that AF recurrence and the need for redo-AF ablation was lower in 51 patients who underwent bariatric surgery before undergoing catheter ablation for AF, with outcomes even surpassing those in non-obese cohorts [111, 112]. This suggests that invasive management of obesity remains an option for patients who are unresponsive to aggressive non-invasive measures, such as structured exercise programs and diet modification alone; however, further meta-analyses or prospective studies are required to consolidate these findings. The LEAF study [113] further reinforces the importance of weight loss pre-procedure in enhancing AF ablation outcomes and also provides emerging evidence for the independent impact of weight-loss drugs on the improved outcomes post-AF ablation; a striking 83% one-year freedom from AF reported in their Liraglutide + risk factor modification group, versus 57% seen in the risk factor modification only group [114].

### 3.6 Hyperlipidemia

Observational studies have shown inconsistent results regarding the association between lower cholesterol and AF risk, with some demonstrating a paradoxical lower risk with higher low density lipoprotein (LDL) levels [115, 116]. The relationship is likely consequential, with a deranged lipid profile increasing the risk of adverse CV events, providing a source to AF substrate [117]. Nonetheless, the exact mechanisms remain unclear, and until further evidence suggests otherwise, hyperlipidemia, at the least, should be managed as part of the overall CV risk [115].

### 3.7 Exercise and Physical Inactivity

Those who live more sedentary lives are at higher risk of AF due to the associated risk of poorer CV health and the accumulation of other co-morbidities, including a higher risk of obesity, high BP, and DM [118]. Lack of physical activity further augments cardiomyopathy, exacerbating the AF substrate. Conversely, moderate regular activity can offset some of the AF risk associated with obesity and attenuate the increased risk of AF with LA enlargement [119]. Cardiorespiratory fitness has been demonstrated to predict arrhythmia recurrence in obese individuals with symptomatic AF, and an improvement in cardiorespiratory fitness augments the beneficial effects of weight loss [120]. Moderate amounts of activity significantly reduce AF risk [121]. There is a reduced risk of AF with moderate-intensity physical activity versus no exercise, which is not seen in low- or high-intensity exercise [122]. A balanced activity approach with a ‘u-shaped’ dose response to exercise is important. Supervised exercise is safe and beneficial to patients with AF. Even short, tailored physical activity programs have proven to reduce AF recurrence, improve LA and ventricular function parameters, improve quality of life, and increase cardiorespiratory fitness in participants with AF [123, 124, 125, 126, 127]. Physical activity as part of a cardiac rehabilitation model after AF ablation, as shown in the CopenHeartRFA trial [128], showed the impact of 12 weeks of physical exercise sessions with four psycho-educational consultations. Cardiorespiratory fitness, measured by VO_2_ max, increased and was maintained at the 12-month follow-up, and a lower proportion of patients had high anxiety at the 24-month follow-up.

### 3.8 Alcohol Consumption

The causal relationship between alcohol consumption and AF is well established, demonstrating a dose-dependent relationship [129] and a more prominent link with acute heavy drinking [130]. Two meta-analyses have further consolidated the linear dose-response relationship between alcohol intake and AF incidence [131, 132]. The Framingham study showed a significant 8% increase in the relative risk in the incidence of AF for each standard drink per day, compared to no alcohol at all [133]. A recent study also found that abstinence from alcohol significantly reduced arrhythmia recurrences at the 6-month follow-up in regular drinkers with AF (both paroxysmal and persistent) who were in sinus rhythm at baseline [134]. Alcohol has been shown to affect the autonomic nervous system, shorten the atrial ERP, and be associated with LA enlargement, all of which contribute to the pathophysiology of AF, as mentioned previously [135, 136]. Studies have also clearly demonstrated the link between alcohol and AF recurrence post-catheter ablation. In a 2016 Japanese study of 1361 patients with paroxysmal AF undergoing their first catheter ablation, AF recurrence post-intervention was noted to be higher in those who consumed alcohol compared to those who were abstinent [137]. In a Chinese cohort of 122 patients undergoing PVI for paroxysmal AF, LA voltage mapping reportedly showed a greater degree of low-voltage zones in those who consumed moderate to high amounts of alcohol, with the procedural success rate being 69.2% and 35.1%, respectively, compared to a success rate of 81.3% in those who abstained from alcohol consumption [138]. In light of the evidence, international guidelines [7, 10] recommend minimizing alcohol consumption, ideally achieving complete abstinence, in individuals with AF, with a stronger emphasis on those pursuing rhythm-control strategies.

### 3.9 Smoking

Numerous prospective cohort studies have previously tried to explore the link between smoking and the increased risk of AF; however, reported results have varied. Results reported from the ARIC study showed that current smoking accounted for about a 10% increase in the incidence of AF [139]. In contrast, other authors have documented an increase in risk of up to 32% in current smokers, with some authors even presenting double this figure [139, 140]. Oxidative stress, an increase in sympathetic tone, and fibrotic changes to the atrial wall have been implicated in the pathological effects of smoking in the development of AF. In a study involving 59 patients with refractory AF undergoing PVI, the diameter of the pulmonary veins and the LA volumes was larger in smokers with an AF recurrence rate of 43% post-ablation, compared to 14% in non-smokers [141]. Though conclusive evidence regarding smoking and AF prevention (post-AF ablation) is lacking at present, smoking cessation is strongly recommended in general, especially in patients with CV disease.

### 3.10 Caffeine Intake

Studies have investigated the relationship between caffeine or coffee consumption and AF risk, collectively challenging previous concerns about the arrhythmogenic potential of caffeine [142]. Cheng *et al*. [143] conducted a dose-response meta-analysis of prospective cohort studies and found that each 300 mg/day increase in habitual caffeine intake was associated with a 6% lower risk of AF, suggesting a potential protective effect. Similarly, Krittanawong *et al*. [144], in a systematic review and meta-analysis, concluded that caffeine or coffee consumption does not increase the risk of new-onset AF and may even offer modest protective benefits. Bodar *et al*. [145], in the Physicians’ Health Study, observed no significant association between coffee consumption and AF incidence in a large cohort of male physicians, with no evidence of a dose-response relationship. Together, these findings reassure that moderate caffeine consumption is not associated with an increased risk of AF and may confer a protective effect, supporting a more nuanced understanding of the impact of caffeine on cardiovascular health.

## 4. Comprehensive Risk Factor Modification Approaches 

While addressing individual risk factors is beneficial, a holistic approach to management has demonstrated superior outcomes. The best demonstration of this was in the ARREST-AF study [110]. Here, a total of 149 consecutive patients undergoing AF ablation with BMI ≥27 kg/m^2^, with at least one cardiac risk factor, were given the choice to receive aggressive risk factor modification (61 participants) versus a cohort of those who declined the intervention (88 participants). The study demonstrated intensive interventions to control obesity, hypertension, DM, hyperlipidemia, and sleep apnea had significantly lower AF recurrence rates than those receiving usual care. Over 12 months, the aggressive risk factor management group showed higher rates of freedom from AF (87% compared to 17.8% in control), with weight loss emerging as a major factor in improving ablation success.

Our CREED-AF study is recruiting and represents a randomized controlled trial designed to evaluate the effectiveness of cardiac prehabilitation, rehabilitation, and patient education on AF risk factors in those undergoing first-time AF ablation [146]. The study aims to determine if these simple interventions can improve cardiorespiratory fitness and explore the impact on clinical factors related to AF ablation, including health-related quality-of-life, AF recurrence and burden, and the requirement for repeat AF ablation; the study defined major adverse cardiovascular events and cost-effectiveness of such an intervention. Furthermore, the study introduces a novel approach by incorporating comprehensive risk factor management in the form of planned prehabilitation (a program of education, risk factor assessment, and exercise pre-procedure), in addition to rehabilitation, into a randomized controlled trial, providing a rigorous evaluation of its potential benefits for patients undergoing AF ablation.

**Fig. 3.**
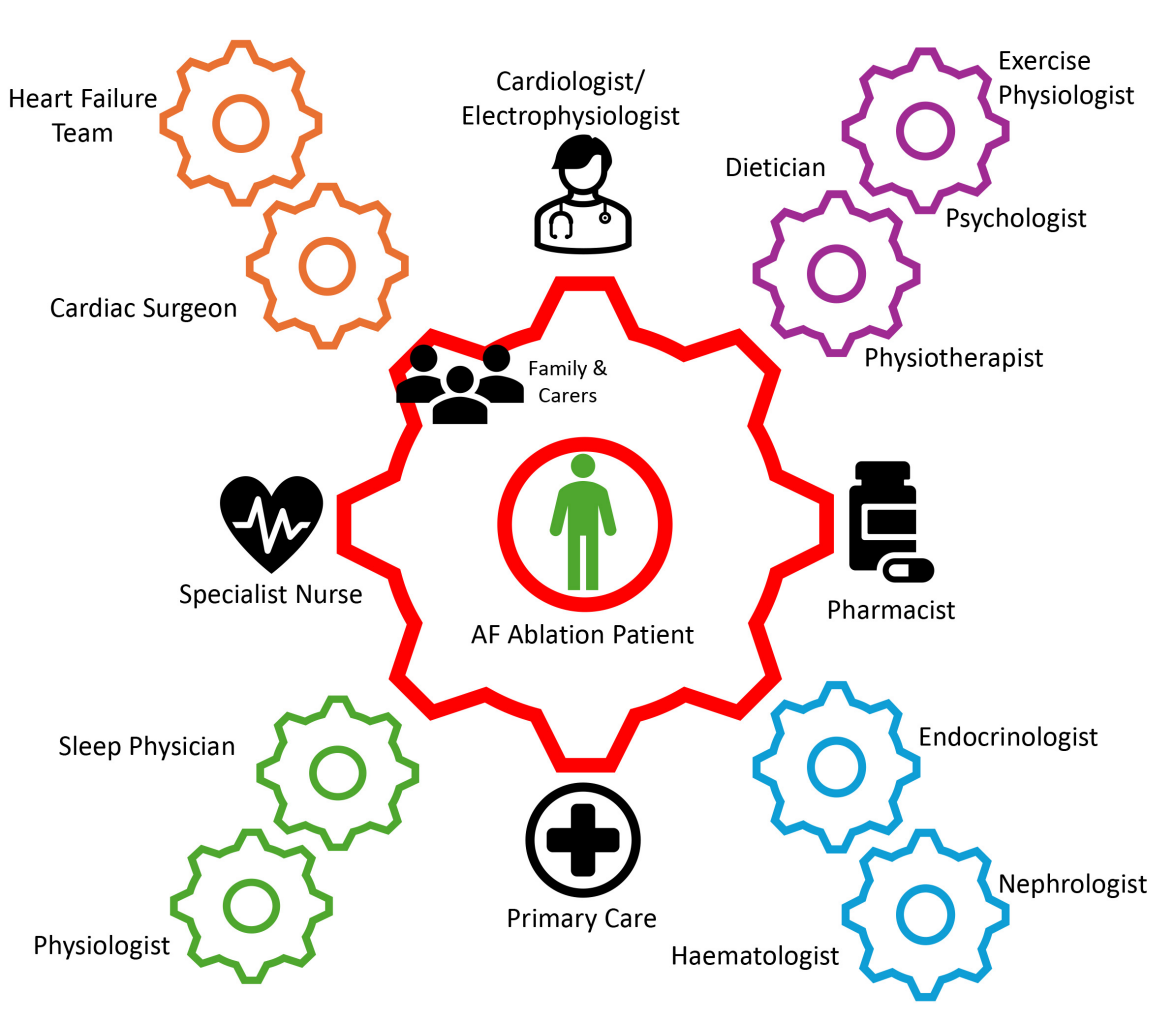
**Demonstration of the multi-disciplinary team ‘hub-and-spoke’ approach to the management of patients undergoing an AF ablation procedure**. AF, atrial fibrillation.

Increasing evidence supports a holistic/integrated AF care approach to each patient, working with them to tackle co-morbidities [7]. Meanwhile, international guidelines call for employing a multidisciplinary-based approach [7, 10]. Though this approach may be resource-intensive, it is preferred over opportunistic methods. Indeed, a ’hub-and-spoke’ model could be suggested, with a central coordinating team including the cardiologists, general practitioners, specialist nurses, and pharmacists, and further involving other healthcare professionals depending on local funding and targeted therapy (see Fig. 3). Multiple models, including multi-disciplinary teams, nurse-led clinics, or cardiologist-led, have been employed and published with mixed results [147, 148, 149, 150]. Hendriks *et al*. [147] demonstrated better outcomes of CV hospitalizations and CV mortality in AF patients with a nurse-led care group compared to usual care. However, the recent RACE 4 trial [148] failed to show the superiority of a nurse-led over usual care approaches but suggested that nurse-led care by an experienced team could be clinically beneficial. Thus, more evidence on these strategies is required [7].

## 5. Conclusions

Effective risk factor management is crucial in treating AF, especially in those undergoing AF ablation, regardless of the approach. A comprehensive approach to managing risk factors, including hypertension, obesity, DM, OSA, HF, and lifestyle choices, such as smoking and excessive alcohol consumption, can significantly reduce AF recurrence and improve long-term prognosis. A summary of the pertinent studies explored in our review is represented in Table 1 (Ref. [59, 60, 61, 71, 72, 73, 93, 94, 95, 110, 111, 112, 113, 128]). Importantly, with AF predicted to become an increasing load on healthcare systems worldwide, any attempts to curtail and enhance the current epidemic will ease and potentially reverse this growing burden. The broad adoption and assessment of risk factor management are essential in this endeavor.

**Table 1.** **Summaries of studies explored demonstrating impact of risk factor modification on AF ablation outcomes**.

Study	Population	Risk factor modification/intervention	Outcomes
Hypertension			
Parkash *et al*., 2017 (SMAC-AF) [59]	AF patients (57% paroxysmal)	Aggressive BP treatment (target <120 mmHg) vs. standard BP treatment (target <140 mmHg)	At 12 months: recurrence of AF/atrial tachycardia/atrial flutter not different to control group (*p* = 0.763)
Pokushalov *et al*., 2012 [60]	AF patients refractory to 2 AAD with drug-resistant hypertension	Renal denervation in addition to PVI vs. PVI alone	At 12 months: intervention group: 69% arrhythmia-free; control group: 29% arrhythmia-free (*p* = 0.033)
Steinberg *et al*., 2020 (ERADICATE-AF) [61]	Paroxysmal AF patients	Renal denervation in addition to PVI vs. PVI alone	At 12 months: intervention group: 72% freedom from AF recurrence; control group: 57% freedom from AF recurrence (*p* = 0.006)
Diabetes mellitus			
Donnellan *et al*., 2019 [71]	AF patients with diabetes (40% paroxysmal)	Pre-procedure HbA1c control <7% vs. poor control	At 26 months: HbA1c control (<7%): 32.4% AF recurrence; HbA1c poor control (>9%): 69% AF recurrence (*p * < 0.0001). A 10% improvement in HbA1c in 12-month pre-ablation period: 2% AF recurrence; HbA1c worsening trend: 91% AF recurrence at 26 months (*p * < 0.0001)
Abu-Qaoud *et al*., 2023 [72]	AF patients with diabetes	Baseline SGLTi use vs. no baseline SGLTi use	SGLTi use: 27.8% event rate; no SGLTi use: 36% event rate (*p * < 0.0001). Composite of cardioversion, new initiation of AAD, or re-do AF ablation at 12-months, but after a 3-month blanking
Zhao *et al*., 2023 [73]	AF patients with diabetes	Baseline SGLTi use vs. no baseline SGLTi use	At 18 months: SGLTi use: 26.8% AF recurrence; no SGLTi use: 39.3% AF recurrence (*p * < 0.007)
Obstructive sleep apnea			
Fein *et al*., 2013 [93]	AF patients (53% persistent AF)	Treatment of OSA vs. non-treatment	At 12 months: with CPAP: 72% arrhythmia-free survival; without CPAP: 37% arrhythmia-free survival (*p* = 0.01)
Patel *et al*., 2010 [94]	AF patients (53% paroxysmal AF)	Treatment of OSA vs. non-treatment	At 32 months: with CPAP: 79% arrhythmia-free survival; without CPAP: 68% arrhythmia-free survival (*p* = 0.001)
Naruse *et al*., 2013 [95]	AF patients (54% paroxysmal AF)	Treatment of OSA vs. non-treatment	At 19 months: with CPAP: 30% AF recurrence; without CPAP: 53% AF recurrence (*p * < 0.01)
Obesity			
Donnellan *et al*., 2019 [111]	AF patients, BMI 41 (39% paroxysmal AF)	Bariatric surgery vs. no bariatric surgery	At 36 months: bariatric surgery group: 20% AF recurrence; no bariatric surgery group: 61% AF recurrence (*p * < 0.0001) At 36 months: bariatric surgery group: 12% repeat procedure; no bariatric surgery group: 41% repeat procedure (*p * < 0.0001)
Donnellan *et al*., 2019 [112]	AF patients, BMI 35 (41% paroxysmal AF)	Bariatric surgery vs. no bariatric surgery vs. non-obese	At 6 months: Group 1: 57% freedom from AF, Group 2: 85% freedom from AF (Fisher’s Test: *p* = 0.085; OLR: *p* = 0.046) (*p * < 0.0001) At 29 months: comparable AF recurrence in the bariatric surgery group (20%) and non-obese group (24.5%); no bariatric surgery group: 55% AF recurrence
Goldberger *et al*., 2023 (LEAF Study) [113]	AF patients, BMI 36 (20% paroxysmal AF)	RFM + Liraglutide or RFM alone	At 6 months: Group 1: 57% freedom from AF, Group 2: 85% freedom from AF (Fisher’s Test: *p* = 0.085; OLR: *p* = 0.046) At 12 months: RFM + Liraglutide: 83% freedom from AF; RFM alone: 57% freedom from AF. Group 1 (<3% weight loss), Group 2 (3–10% weight loss)
Exercise/cardiac rehabilitation			
Risom *et al*., 2020 (CopenHeartRFA) [128]	AF patients (72% paroxysmal)	12 weeks of cardiac rehabilitation vs. usual care	VO_2_ max increased in the cardiac rehabilitation group vs. controls, but no significant difference in mental health or other SF-36 components at 4- and 12-month follow-ups
Comprehensive risk factor management			
Pathak *et al*., 2014 (ARREST-AF) [110]	AF patients, BMI >27	Aggressive comprehensive RFM vs. usual care	At 41 months: RFM group: reduced AF-symptom burden (*p * < 0.001). RFM group: 87% arrhythmia-free survival; control group: 17% arrhythmia-free survival (*p * < 0.001)

AAD, anti-arrhythmic drugs; AF, atrial fibrillation; BP, blood pressure; CPAP, continuous positive airway pressure; HbA1c, glycated haemoglobin; OLR, ordinal logistic regression; OSA, obstructive sleep apnea; PVI, pulmonary vein isolation; RFM, risk factor management or risk factor modification; SF-36, 36-item short form survey; SGLTi, sodium glucose co-transporter 2 inhibitor; BMI, body mass index.

## References

[1] Lippi G, Sanchis-Gomar F, Cervellin G (2021). Global epidemiology of atrial fibrillation: An increasing epidemic and public health challenge. *International Journal of Stroke: Official Journal of the International Stroke Society*.

[2] Roth GA, Mensah GA, Johnson CO, Addolorato G, Ammirati E, Baddour LM (2024). Global Burden of Cardiovascular Diseases and Risk Factors, 1990–2019: Update From the GBD 2019 Study. *Journal of the American College of Cardiology*.

[3] Schnabel RB, Yin X, Gona P, Larson MG, Beiser AS, McManus DD (2015). 50 year trends in atrial fibrillation prevalence, incidence, risk factors, and mortality in the Framingham Heart Study: a cohort study. *Lancet (London, England)*.

[4] Gallagher C, Hendriks JM, Giles L, Karnon J, Pham C, Elliott AD (2019). Increasing trends in hospitalisations due to atrial fibrillation in Australia from 1993 to 2013. *Heart (British Cardiac Society)*.

[5] Chugh SS, Havmoeller R, Narayanan K, Singh D, Rienstra M, Benjamin EJ (2014). Worldwide epidemiology of atrial fibrillation: a Global Burden of Disease 2010 Study. *Circulation*.

[6] Colilla S, Crow A, Petkun W, Singer DE, Simon T, Liu X (2013). Estimates of current and future incidence and prevalence of atrial fibrillation in the U.S. adult population. *The American Journal of Cardiology*.

[7] Van Gelder IC, Rienstra M, Bunting KV, Casado-Arroyo R, Caso V, Crijns HJGM (2024). 2024 ESC Guidelines for the management of atrial fibrillation developed in collaboration with the European Association for Cardio-Thoracic Surgery (EACTS). *European Heart Journal*.

[8] Camm AJ, Kirchhof P, Lip GYH, Schotten U, European Heart Rhythm Association, European Association for Cardio-Thoracic Surgery (2010). Guidelines for the management of atrial fibrillation: the Task Force for the Management of Atrial Fibrillation of the European Society of Cardiology (ESC). *European Heart Journal*.

[9] January CT, Wann LS, Alpert JS, Calkins H, Cigarroa JE, Cleveland JC (2014). 2014 AHA/ACC/HRS guideline for the management of patients with atrial fibrillation: a report of the American College of Cardiology/American Heart Association Task Force on practice guidelines and the Heart Rhythm Society. *Circulation*.

[10] Writing Committee Members, Joglar JA, Chung MK, Armbruster AL, Benjamin EJ, Chyou JY (2024). 2023 ACC/AHA/ACCP/HRS Guideline for the Diagnosis and Management of Atrial Fibrillation: A Report of the American College of Cardiology/American Heart Association Joint Committee on Clinical Practice Guidelines. *Journal of the American College of Cardiology*.

[11] Middeldorp ME, Pathak RK, Lau DH, Sanders P (2019). PREVEntion and regReSsive Effect of weight-loss and risk factor modification on Atrial Fibrillation: the REVERSE-AF study-Authors’ reply. *Europace*.

[12] Stout K, Almerstani M, Adomako R, Shin D, Aroudaky A, Tandon H (2023). Prevalence and Impact of Poorly Controlled Modifiable Risk Factors Among Patients Who Underwent Atrial Fibrillation Ablation. *The American Journal of Cardiology*.

[13] Tzeis S, Gerstenfeld EP, Kalman J, Saad E, Shamloo AS, Andrade JG (2024). 2024 European Heart Rhythm Association/Heart Rhythm Society/Asia Pacific Heart Rhythm Society/Latin American Heart Rhythm Society expert consensus statement on catheter and surgical ablation of atrial fibrillation. *Journal of Interventional Cardiac Electrophysiology*.

[14] Wyse DG, Waldo AL, DiMarco JP, Domanski MJ, Rosenberg Y, Schron EB (2002). A comparison of rate control and rhythm control in patients with atrial fibrillation. *The New England Journal of Medicine*.

[15] van Gelder IC, Hagens VE, Kingma JH, Bosker HA, Kamp O, Kingma T (2002). Rate control versus electrical cardioversion for atrial fibrillation: A randomised comparison of two treatment strategies concerning morbidity, mortality, quality of life and cost-benefit - the RACE study design. *Netherlands Heart Journal: Monthly Journal of the Netherlands Society of Cardiology and the Netherlands Heart Foundation*.

[16] Packer DL, Mark DB, Robb RA, Monahan KH, Bahnson TD, Poole JE (2019). Effect of Catheter Ablation vs Antiarrhythmic Drug Therapy on Mortality, Stroke, Bleeding, and Cardiac Arrest Among Patients With Atrial Fibrillation: The CABANA Randomized Clinical Trial. *JAMA*.

[17] Terricabras M, Mantovan R, Jiang CY, Betts TR, Chen J, Deisenhofer I (2020). Association Between Quality of Life and Procedural Outcome After Catheter Ablation for Atrial Fibrillation: A Secondary Analysis of a Randomized Clinical Trial. *JAMA Network Open*.

[18] Mazetto RA, Antunes V, Bulhões E, Defante M, Balieiro C, Ferreira A (2024). Effect of catheter ablation versus medical therapy on mental health and quality of life in patients with atrial fibrillation: a systematic review and meta-analysis of randomized controlled trials. *Journal of Interventional Cardiac Electrophysiology*.

[19] Kirchhof P, Camm AJ, Goette A, Brandes A, Eckardt L, Elvan A (2020). Early Rhythm-Control Therapy in Patients with Atrial Fibrillation. *The New England Journal of Medicine*.

[20] Wazni OM, Marrouche NF, Martin DO, Verma A, Bhargava M, Saliba W (2005). Radiofrequency ablation vs antiarrhythmic drugs as first-line treatment of symptomatic atrial fibrillation: a randomized trial. *JAMA*.

[21] Jaïs P, Cauchemez B, Macle L, Daoud E, Khairy P, Subbiah R (2008). Catheter ablation versus antiarrhythmic drugs for atrial fibrillation: the A4 study. *Circulation*.

[22] Wilber DJ, Pappone C, Neuzil P, De Paola A, Marchlinski F, Natale A (2010). Comparison of antiarrhythmic drug therapy and radiofrequency catheter ablation in patients with paroxysmal atrial fibrillation: a randomized controlled trial. *JAMA*.

[23] Packer DL, Kowal RC, Wheelan KR, Irwin JM, Champagne J, Guerra PG (2013). Cryoballoon ablation of pulmonary veins for paroxysmal atrial fibrillation: first results of the North American Arctic Front (STOP AF) pivotal trial. *Journal of the American College of Cardiology*.

[24] Mont L, Bisbal F, Hernández-Madrid A, Pérez-Castellano N, Viñolas X, Arenal A (2014). Catheter ablation vs. antiarrhythmic drug treatment of persistent atrial fibrillation: a multicentre, randomized, controlled trial (SARA study). *European Heart Journal*.

[25] Morillo CA, Verma A, Connolly SJ, Kuck KH, Nair GM, Champagne J (2014). Radiofrequency ablation vs antiarrhythmic drugs as first-line treatment of paroxysmal atrial fibrillation (RAAFT-2): a randomized trial. *JAMA*.

[26] Wazni OM, Dandamudi G, Sood N, Hoyt R, Tyler J, Durrani S (2021). Cryoballoon Ablation as Initial Therapy for Atrial Fibrillation. *The New England Journal of Medicine*.

[27] Andrade JG, Wells GA, Deyell MW, Bennett M, Essebag V, Champagne J (2021). Cryoablation or Drug Therapy for Initial Treatment of Atrial Fibrillation. *The New England Journal of Medicine*.

[28] Kuniss M, Pavlovic N, Velagic V, Hermida JS, Healey S, Arena G (2021). Cryoballoon ablation vs. antiarrhythmic drugs: first-line therapy for patients with paroxysmal atrial fibrillation. *Europace*.

[29] Andrade JG, Deyell MW, Macle L, Wells GA, Bennett M, Essebag V (2023). Progression of Atrial Fibrillation after Cryoablation or Drug Therapy. *The New England Journal of Medicine*.

[30] Andrade JG (2024). Ablation or drug therapy for initial atrial fibrillation. *Annals of Cardiothoracic Surgery*.

[31] Han FT, Bartus K, Lakkireddy D, Rojas F, Bednarek J, Kapelak B (2014). The effects of LAA ligation on LAA electrical activity. *Heart Rhythm*.

[32] Fink T, Schlüter M, Heeger CH, Lemes C, Maurer T, Reissmann B (2017). Stand-Alone Pulmonary Vein Isolation Versus Pulmonary Vein Isolation With Additional Substrate Modification as Index Ablation Procedures in Patients With Persistent and Long-Standing Persistent Atrial Fibrillation: The Randomized Alster-Lost-AF Trial (Ablation at St. Georg Hospital for Long-Standing Persistent Atrial Fibrillation). *Circulation. Arrhythmia and Electrophysiology*.

[33] Di Biase L, Burkhardt JD, Mohanty P, Sanchez J, Mohanty S, Horton R (2010). Left atrial appendage: an underrecognized trigger site of atrial fibrillation. *Circulation*.

[34] Phan QT, Shin SY, Cho IS, Lee WS, Won H, Sharmin S (2019). Impact of left atrial appendage closure on cardiac functional and structural remodeling: A difference-in-difference analysis of propensity score matched samples. *Cardiology Journal*.

[35] Lakkireddy DR, Wilber DJ, Mittal S, Tschopp D, Ellis CR, Rasekh A (2024). Pulmonary Vein Isolation With or Without Left Atrial Appendage Ligation in Atrial Fibrillation: The aMAZE Randomized Clinical Trial. *JAMA*.

[36] Ganesan AN, Shipp NJ, Brooks AG, Kuklik P, Lau DH, Lim HS (2013). Long-term outcomes of catheter ablation of atrial fibrillation: a systematic review and meta-analysis. *Journal of the American Heart Association*.

[37] Miyazaki S, Kuwahara T, Kobori A, Takahashi Y, Takei A, Sato A (2011). Long-term clinical outcome of extensive pulmonary vein isolation-based catheter ablation therapy in patients with paroxysmal and persistent atrial fibrillation. *Heart (British Cardiac Society)*.

[38] Steinberg JS, Palekar R, Sichrovsky T, Arshad A, Preminger M, Musat D (2014). Very long-term outcome after initially successful catheter ablation of atrial fibrillation. *Heart Rhythm*.

[39] Takigawa M, Takahashi A, Kuwahara T, Okubo K, Takahashi Y, Watari Y (2014). Long-term follow-up after catheter ablation of paroxysmal atrial fibrillation: the incidence of recurrence and progression of atrial fibrillation. *Circulation. Arrhythmia and Electrophysiology*.

[40] Berman AE, Kabiri M, Wei T, Galvain T, Sha Q, Kuck KH (2023). Economic and Health Value of Delaying Atrial Fibrillation Progression Using Radiofrequency Catheter Ablation. *Circulation. Arrhythmia and Electrophysiology*.

[41] Teh AW, Kistler PM, Lee G, Medi C, Heck PM, Spence SJ (2012). Long-term effects of catheter ablation for lone atrial fibrillation: progressive atrial electroanatomic substrate remodeling despite successful ablation. *Heart Rhythm*.

[42] Benjamin EJ, Wolf PA, D’Agostino RB, Silbershatz H, Kannel WB, Levy D (1998). Impact of atrial fibrillation on the risk of death: the Framingham Heart Study. *Circulation*.

[43] Lau DH, Mackenzie L, Kelly DJ, Psaltis PJ, Worthington M, Rajendram A (2010). Short-term hypertension is associated with the development of atrial fibrillation substrate: a study in an ovine hypertensive model. *Heart Rhythm*.

[44] Lau DH, Mackenzie L, Kelly DJ, Psaltis PJ, Brooks AG, Worthington M (2010). Hypertension and atrial fibrillation: evidence of progressive atrial remodeling with electrostructural correlate in a conscious chronically instrumented ovine model. *Heart Rhythm*.

[45] McEvoy JW, McCarthy CP, Bruno RM, Brouwers S, Canavan MD, Ceconi C (2024). 2024 ESC Guidelines for the management of elevated blood pressure and hypertension. *European Heart Journal*.

[46] Okin PM, Hille DA, Larstorp ACK, Wachtell K, Kjeldsen SE, Dahlöf B (2015). Effect of lower on-treatment systolic blood pressure on the risk of atrial fibrillation in hypertensive patients. *Hypertension (Dallas, Tex.: 1979)*.

[47] Kim D, Yang PS, Kim TH, Jang E, Shin H, Kim HY (2018). Ideal Blood Pressure in Patients With Atrial Fibrillation. *Journal of the American College of Cardiology*.

[48] Wachtell K, Lehto M, Gerdts E, Olsen MH, Hornestam B, Dahlöf B (2005). Angiotensin II receptor blockade reduces new-onset atrial fibrillation and subsequent stroke compared to atenolol: the Losartan Intervention For End Point Reduction in Hypertension (LIFE) study. *Journal of the American College of Cardiology*.

[49] Schmieder RE, Kjeldsen SE, Julius S, McInnes GT, Zanchetti A, Hua TA (2008). Reduced incidence of new-onset atrial fibrillation with angiotensin II receptor blockade: the VALUE trial. *Journal of Hypertension*.

[50] Galzerano D, Di Michele S, Paolisso G, Tuccillo B, Lama D, Carbotta S (2012). A multicentre, randomized study of telmisartan versus carvedilol for prevention of atrial fibrillation recurrence in hypertensive patients. *Journal of the Renin-angiotensin-aldosterone System: JRAAS*.

[51] Du H, Fan J, Ling Z, Woo K, Su L, Chen S (2013). Effect of nifedipine versus telmisartan on prevention of atrial fibrillation recurrence in hypertensive patients. *Hypertension (Dallas, Tex.: 1979)*.

[52] Casaclang-Verzosa G, Gersh BJ, Tsang TSM (2008). Structural and functional remodeling of the left atrium: clinical and therapeutic implications for atrial fibrillation. *Journal of the American College of Cardiology*.

[53] Berruezo A, Tamborero D, Mont L, Benito B, Tolosana JM, Sitges M (2007). Pre-procedural predictors of atrial fibrillation recurrence after circumferential pulmonary vein ablation. *European Heart Journal*.

[54] Letsas KP, Weber R, Bürkle G, Mihas CC, Minners J, Kalusche D (2009). Pre-ablative predictors of atrial fibrillation recurrence following pulmonary vein isolation: the potential role of inflammation. *Europace*.

[55] Khaykin Y, Oosthuizen R, Zarnett L, Essebag V, Parkash R, Seabrook C (2011). Clinical predictors of arrhythmia recurrences following pulmonary vein antrum isolation for atrial fibrillation: predicting arrhythmia recurrence post-PVAI. *Journal of Cardiovascular Electrophysiology*.

[56] Santoro F, Di Biase L, Trivedi C, Burkhardt JD, Paoletti Perini A, Sanchez J (2015). Impact of Uncontrolled Hypertension on Atrial Fibrillation Ablation Outcome. *JACC. Clinical Electrophysiology*.

[57] Kamioka M, Hijioka N, Matsumoto Y, Nodera M, Kaneshiro T, Suzuki H (2018). Uncontrolled blood pressure affects atrial remodeling and adverse clinical outcome in paroxysmal atrial fibrillation. *Pacing and Clinical Electrophysiology: PACE*.

[58] Zylla MM, Hochadel M, Andresen D, Brachmann J, Eckardt L, Hoffmann E (2020). Ablation of Atrial Fibrillation in Patients with Hypertension-An Analysis from the German Ablation Registry. *Journal of Clinical Medicine*.

[59] Parkash R, Wells GA, Sapp JL, Healey JS, Tardif JC, Greiss I (2017). Effect of Aggressive Blood Pressure Control on the Recurrence of Atrial Fibrillation After Catheter Ablation: A Randomized, Open-Label Clinical Trial (SMAC-AF [Substrate Modification With Aggressive Blood Pressure Control]). *Circulation*.

[60] Pokushalov E, Romanov A, Corbucci G, Artyomenko S, Baranova V, Turov A (2012). A randomized comparison of pulmonary vein isolation with versus without concomitant renal artery denervation in patients with refractory symptomatic atrial fibrillation and resistant hypertension. *Journal of the American College of Cardiology*.

[61] Steinberg JS, Shabanov V, Ponomarev D, Losik D, Ivanickiy E, Kropotkin E (2020). Effect of Renal Denervation and Catheter Ablation vs Catheter Ablation Alone on Atrial Fibrillation Recurrence Among Patients With Paroxysmal Atrial Fibrillation and Hypertension: The ERADICATE-AF Randomized Clinical Trial. *JAMA*.

[62] Aune D, Feng T, Schlesinger S, Janszky I, Norat T, Riboli E (2018). Diabetes mellitus, blood glucose and the risk of atrial fibrillation: A systematic review and meta-analysis of cohort studies. *Journal of Diabetes and its Complications*.

[63] Huxley RR, Alonso A, Lopez FL, Filion KB, Agarwal SK, Loehr LR (2012). Type 2 diabetes, glucose homeostasis and incident atrial fibrillation: the Atherosclerosis Risk in Communities study. *Heart (British Cardiac Society)*.

[64] Seyed Ahmadi S, Svensson AM, Pivodic A, Rosengren A, Lind M (2020). Risk of atrial fibrillation in persons with type 2 diabetes and the excess risk in relation to glycaemic control and renal function: a Swedish cohort study. *Cardiovascular Diabetology*.

[65] Benjamin EJ, Levy D, Vaziri SM, D’Agostino RB, Belanger AJ, Wolf PA (1994). Independent risk factors for atrial fibrillation in a population-based cohort. The Framingham Heart Study. *JAMA*.

[66] Lorenzo-Almorós A, Casado Cerrada J, Álvarez-Sala Walther LA, Méndez Bailón M, Lorenzo González Ó (2023). Atrial Fibrillation and Diabetes Mellitus: Dangerous Liaisons or Innocent Bystanders?. *Journal of Clinical Medicine*.

[67] Wang A, Truong T, Black-Maier E, Green C, Campbell KB, Barnett AS (2020). Catheter ablation of atrial fibrillation in patients with diabetes mellitus. *Heart Rhythm O2*.

[68] Creta A, Providência R, Adragão P, de Asmundis C, Chun J, Chierchia G (2020). Impact of Type-2 Diabetes Mellitus on the Outcomes of Catheter Ablation of Atrial Fibrillation (European Observational Multicentre Study). *The American Journal of Cardiology*.

[69] Bogossian H, Frommeyer G, Brachmann J, Lewalter T, Hoffmann E, Kuck KH (2016). Catheter ablation of atrial fibrillation and atrial flutter in patients with diabetes mellitus: Who benefits and who does not? Data from the German ablation registry. *International Journal of Cardiology*.

[70] Anselmino M, Matta M, D’ascenzo F, Pappone C, Santinelli V, Bunch TJ (2015). Catheter ablation of atrial fibrillation in patients with diabetes mellitus: a systematic review and meta-analysis. *Europace*.

[71] Donnellan E, Aagaard P, Kanj M, Jaber W, Elshazly M, Hoosien M (2019). Association Between Pre-Ablation Glycemic Control and Outcomes Among Patients With Diabetes Undergoing Atrial Fibrillation Ablation. *JACC. Clinical Electrophysiology*.

[72] Abu-Qaoud MR, Kumar A, Tarun T, Abraham S, Ahmad J, Khadke S (2023). Impact of SGLT2 Inhibitors on AF Recurrence After Catheter Ablation in Patients With Type 2 Diabetes. *JACC. Clinical Electrophysiology*.

[73] Zhao Z, Jiang C, He L, Zheng S, Wang Y, Gao M (2023). Impact of Sodium-Glucose Cotransporter 2 Inhibitor on Recurrence After Catheter Ablation for Atrial Fibrillation in Patients With Diabetes: A Propensity-Score Matching Study and Meta-Analysis. *J Am Heart Assoc*.

[74] Carlisle MA, Fudim M, DeVore AD, Piccini JP (2019). Heart Failure and Atrial Fibrillation, Like Fire and Fury. *JACC. Heart Failure*.

[75] Santhanakrishnan R, Wang N, Larson MG, Magnani JW, McManus DD, Lubitz SA (2016). Atrial Fibrillation Begets Heart Failure and Vice Versa: Temporal Associations and Differences in Preserved Versus Reduced Ejection Fraction. *Circulation*.

[76] Zannad F, McMurray JJV, Krum H, van Veldhuisen DJ, Swedberg K, Shi H (2011). Eplerenone in patients with systolic heart failure and mild symptoms. *The New England Journal of Medicine*.

[77] McMurray JJV, Packer M, Desai AS, Gong J, Lefkowitz MP, Rizkala AR (2014). Angiotensin-neprilysin inhibition versus enalapril in heart failure. *The New England Journal of Medicine*.

[78] Bhatt DL, Szarek M, Steg PG, Cannon CP, Leiter LA, McGuire DK (2021). Sotagliflozin in Patients with Diabetes and Recent Worsening Heart Failure. *The New England Journal of Medicine*.

[79] Anker SD, Butler J, Filippatos G, Ferreira JP, Bocchi E, Böhm M (2021). Empagliflozin in Heart Failure with a Preserved Ejection Fraction. *The New England Journal of Medicine*.

[80] Solomon SD, McMurray JJV, Claggett B, De Boer RA, DeMets D, Hernandez AF (2022). Dapagliflozin in Heart Failure with Mildly Reduced or Preserved Ejection Fraction. *The New England Journal of Medicine*.

[81] Pandey AK, Okaj I, Kaur H, Belley-Cote EP, Wang J, Oraii A (2021). Sodium-Glucose Co-Transporter Inhibitors and Atrial Fibrillation: A Systematic Review and Meta-Analysis of Randomized Controlled Trials. *Journal of the American Heart Association*.

[82] Lim VG, He H, Lachlan T, Ng GA, Kyrou I, Randeva HS (2022). Impact of sodium-glucose co-transporter inhibitors on cardiac autonomic function and mortality: no time to die. *Europace*.

[83] Khan MN, Jaïs P, Cummings J, Di Biase L, Sanders P, Martin DO (2008). Pulmonary-vein isolation for atrial fibrillation in patients with heart failure. *The New England Journal of Medicine*.

[84] Jones DG, Haldar SK, Hussain W, Sharma R, Francis DP, Rahman-Haley SL (2013). A randomized trial to assess catheter ablation versus rate control in the management of persistent atrial fibrillation in heart failure. *Journal of the American College of Cardiology*.

[85] Di Biase L, Mohanty P, Mohanty S, Santangeli P, Trivedi C, Lakkireddy D (2016). Ablation Versus Amiodarone for Treatment of Persistent Atrial Fibrillation in Patients With Congestive Heart Failure and an Implanted Device: Results From the AATAC Multicenter Randomized Trial. *Circulation*.

[86] Marrouche NF, Brachmann J, Andresen D, Siebels J, Boersma L, Jordaens L (2018). Catheter Ablation for Atrial Fibrillation with Heart Failure. *The New England Journal of Medicine*.

[87] Sohns C, Fox H, Marrouche NF, Crijns HJGM, Costard-Jaeckle A, Bergau L (2023). Catheter Ablation in End-Stage Heart Failure with Atrial Fibrillation. *The New England Journal of Medicine*.

[88] Rienstra M, Hobbelt AH, Alings M, Tijssen JGP, Smit MD, Brügemann J (2018). Targeted therapy of underlying conditions improves sinus rhythm maintenance in patients with persistent atrial fibrillation: results of the RACE 3 trial. *European Heart Journal*.

[89] Linz D, Schotten U, Neuberger HR, Böhm M, Wirth K (2011). Negative tracheal pressure during obstructive respiratory events promotes atrial fibrillation by vagal activation. *Heart Rhythm*.

[90] Holtstrand Hjälm H, Fu M, Hansson P, Zhong Y, Caidahl K, Mandalenakis Z (2018). Association between left atrial enlargement and obstructive sleep apnea in a general population of 71-year-old men. *Journal of Sleep Research*.

[91] Iwasaki YK, Kato T, Xiong F, Shi YF, Naud P, Maguy A (2014). Atrial fibrillation promotion with long-term repetitive obstructive sleep apnea in a rat model. *Journal of the American College of Cardiology*.

[92] Congrete S, Bintvihok M, Thongprayoon C, Bathini T, Boonpheng B, Sharma K (2018). Effect of obstructive sleep apnea and its treatment of atrial fibrillation recurrence after radiofrequency catheter ablation: A meta-analysis. *Journal of Evidence-based Medicine*.

[93] Fein AS, Shvilkin A, Shah D, Haffajee CI, Das S, Kumar K (2013). Treatment of obstructive sleep apnea reduces the risk of atrial fibrillation recurrence after catheter ablation. *Journal of the American College of Cardiology*.

[94] Patel D, Mohanty P, Di Biase L, Shaheen M, Lewis WR, Quan K (2010). Safety and efficacy of pulmonary vein antral isolation in patients with obstructive sleep apnea: the impact of continuous positive airway pressure. *Circulation. Arrhythmia and Electrophysiology*.

[95] Naruse Y, Tada H, Satoh M, Yanagihara M, Tsuneoka H, Hirata Y (2013). Concomitant obstructive sleep apnea increases the recurrence of atrial fibrillation following radiofrequency catheter ablation of atrial fibrillation: clinical impact of continuous positive airway pressure therapy. *Heart Rhythm*.

[96] He H, Lachlan T, Chandan N, Lim VG, Kimani P, Ng GA (2023). Obstructive Sleep Apnoea and Cardiac Arrhythmias (OSCA) trial: a nested cohort study using injectable loop recorders and Holter monitoring in patients with obstructive sleep apnoea. *BMJ Open*.

[97] Al-Kaisey AM, Kalman JM (2021). Obesity and Atrial Fibrillation: Epidemiology, Pathogenesis and Effect of Weight Loss. *Arrhythmia & Electrophysiology Review*.

[98] Chamberlain AM, Agarwal SK, Folsom AR, Soliman EZ, Chambless LE, Crow R (2011). A clinical risk score for atrial fibrillation in a biracial prospective cohort (from the Atherosclerosis Risk in Communities [ARIC] study). *The American Journal of Cardiology*.

[99] Winkle RA, Mead RH, Engel G, Kong MH, Fleming W, Salcedo J (2017). Impact of obesity on atrial fibrillation ablation: Patient characteristics, long-term outcomes, and complications. *Heart Rhythm*.

[100] Mahajan R, Lau DH, Brooks AG, Shipp NJ, Wood JPM, Manavis J (2021). Atrial Fibrillation and Obesity: Reverse Remodeling of Atrial Substrate With Weight Reduction. *JACC. Clinical Electrophysiology*.

[101] Abed HS, Samuel CS, Lau DH, Kelly DJ, Royce SG, Alasady M (2013). Obesity results in progressive atrial structural and electrical remodeling: implications for atrial fibrillation. *Heart Rhythm*.

[102] Munger TM, Dong YX, Masaki M, Oh JK, Mankad SV, Borlaug BA (2012). Electrophysiological and hemodynamic characteristics associated with obesity in patients with atrial fibrillation. *Journal of the American College of Cardiology*.

[103] Mahajan R, Nelson A, Pathak RK, Middeldorp ME, Wong CX, Twomey DJ (2018). Electroanatomical Remodeling of the Atria in Obesity: Impact of Adjacent Epicardial Fat. *JACC. Clinical Electrophysiology*.

[104] Wong CX, Sullivan T, Sun MT, Mahajan R, Pathak RK, Middeldorp M (2015). Obesity and the Risk of Incident, Post-Operative, and Post-Ablation Atrial Fibrillation: A Meta-Analysis of 626,603 Individuals in 51 Studies. *JACC. Clinical Electrophysiology*.

[105] Tedrow UB, Conen D, Ridker PM, Cook NR, Koplan BA, Manson JE (2010). The long- and short-term impact of elevated body mass index on the risk of new atrial fibrillation the WHS (women’s health study). *Journal of the American College of Cardiology*.

[106] Wang TJ, Parise H, Levy D, D’Agostino RB, Wolf PA, Vasan RS (2004). Obesity and the Risk of New-Onset Atrial Fibrillation. *JAMA*.

[107] Ueno K, Kaneko H, Kamiya K, Itoh H, Okada A, Suzuki Y (2022). Relationship of normal-weight central obesity with the risk for heart failure and atrial fibrillation: analysis of a nationwide health check-up and claims database. *European Heart Journal Open*.

[108] Zhao M, Song L, Zhao Q, Chen Y, Li B, Xie Z (2022). Elevated levels of body mass index and waist circumference, but not high variability, are associated with an increased risk of atrial fibrillation. *BMC Medicine*.

[109] Pathak RK, Middeldorp ME, Meredith M, Mehta AB, Mahajan R, Wong CX (2015). Long-Term Effect of Goal-Directed Weight Management in an Atrial Fibrillation Cohort: A Long-Term Follow-Up Study (LEGACY). *Journal of the American College of Cardiology*.

[110] Pathak RK, Middeldorp ME, Lau DH, Mehta AB, Mahajan R, Twomey D (2014). Aggressive risk factor reduction study for atrial fibrillation and implications for the outcome of ablation: the ARREST-AF cohort study. *Journal of the American College of Cardiology*.

[111] Donnellan E, Wazni OM, Kanj M, Baranowski B, Cremer P, Harb S (2019). Association between pre-ablation bariatric surgery and atrial fibrillation recurrence in morbidly obese patients undergoing atrial fibrillation ablation. *Europace*.

[112] Donnellan E, Wazni O, Kanj M, Hussein A, Baranowski B, Lindsay B (2019). Outcomes of Atrial Fibrillation Ablation in Morbidly Obese Patients Following Bariatric Surgery Compared With a Nonobese Cohort. *Circulation. Arrhythmia and Electrophysiology*.

[113] Goldberger JJ, Mitrani RD, Baez-Garcia C, Blandon C, Zaatari G, Velasquez AH (2023). LB-456089-1 PRE-ABLATION WEIGHT LOSS AS A PREDICTOR OF ATRIAL FIBRILLATION ABLATION OUTCOME IN THE LIRAGLUTIDE EFFECT ON ATRIAL FIBRILLATION (LEAF) STUDY. *Heart Rhythm*.

[114] Hilton L (2023). AHA Scientific Sessions Feature Miller School’s Pioneering Research. https://news.med.miami.edu/leaf-atrial-fibrillation/.

[115] Sagris D, Harrison SL, Lip GYH (2022). Lipids and atrial fibrillation: New insights into a paradox. *PLoS Medicine*.

[116] Ding WY, Protty MB, Davies IG, Lip GYH (2022). Relationship between lipoproteins, thrombosis, and atrial fibrillation. *Cardiovascular Research*.

[117] Liang F, Wang Y (2021). Coronary heart disease and atrial fibrillation: a vicious cycle. *American Journal of Physiology. Heart and Circulatory Physiology*.

[118] Park JH, Moon JH, Kim HJ, Kong MH, Oh YH (2020). Sedentary Lifestyle: Overview of Updated Evidence of Potential Health Risks. *Korean Journal of Family Medicine*.

[119] Heitmann KA, Løchen ML, Stylidis M, Hopstock LA, Schirmer H, Morseth B (2022). Associations between physical activity, left atrial size and incident atrial fibrillation: the Tromsø Study 1994–2016. *Open Heart*.

[120] Pathak RK, Elliott A, Middeldorp ME, Meredith M, Mehta AB, Mahajan R (2015). Impact of CARDIOrespiratory FITness on Arrhythmia Recurrence in Obese Individuals With Atrial Fibrillation: The CARDIO-FIT Study. *Journal of the American College of Cardiology*.

[121] Drca N, Wolk A, Jensen-Urstad M, Larsson SC (2015). Physical activity is associated with a reduced risk of atrial fibrillation in middle-aged and elderly women. *Heart (British Cardiac Society)*.

[122] Mozaffarian D, Furberg CD, Psaty BM, Siscovick D (2008). Physical activity and incidence of atrial fibrillation in older adults: the cardiovascular health study. *Circulation*.

[123] Hegbom F, Stavem K, Sire S, Heldal M, Orning OM, Gjesdal K (2007). Effects of short-term exercise training on symptoms and quality of life in patients with chronic atrial fibrillation. *International Journal of Cardiology*.

[124] Osbak PS, Mourier M, Kjaer A, Henriksen JH, Kofoed KF, Jensen GB (2011). A randomized study of the effects of exercise training on patients with atrial fibrillation. *American Heart Journal*.

[125] Malmo V, Nes BM, Amundsen BH, Tjonna AE, Stoylen A, Rossvoll O (2016). Aerobic Interval Training Reduces the Burden of Atrial Fibrillation in the Short Term: A Randomized Trial. *Circulation*.

[126] Oesterle A, Giancaterino S, Van Noord MG, Pellegrini CN, Fan D, Srivatsa UN (2022). Effects of Supervised Exercise Training on Atrial Fibrillation: A META-ANALYSIS OF RANDOMIZED CONTROLLED TRIALS. *Journal of Cardiopulmonary Rehabilitation and Prevention*.

[127] Elliott AD, Verdicchio CV, Mahajan R, Middeldorp ME, Gallagher C, Mishima RS (2023). An Exercise and Physical Activity Program in Patients With Atrial Fibrillation: The ACTIVE-AF Randomized Controlled Trial. *JACC. Clinical Electrophysiology*.

[128] Risom SS, Zwisler AD, Sibilitz KL, Rasmussen TB, Taylor RS, Thygesen LC (2020). Cardiac Rehabilitation for Patients Treated for Atrial Fibrillation With Ablation Has Long-Term Effects: 12-and 24-Month Follow-up Results From the Randomized CopenHeartRFA Trial. *Archives of Physical Medicine and Rehabilitation*.

[129] Gallagher C, Hendriks JML, Elliott AD, Wong CX, Rangnekar G, Middeldorp ME (2017). Alcohol and incident atrial fibrillation - A systematic review and meta-analysis. *International Journal of Cardiology*.

[130] Ettinger PO, Wu CF, De La Cruz C, Weisse AB, Ahmed SS, Regan TJ (1978). Arrhythmias and the “Holiday Heart”: alcohol-associated cardiac rhythm disorders. *American Heart Journal*.

[131] Kodama S, Saito K, Tanaka S, Horikawa C, Saito A, Heianza Y (2011). Alcohol consumption and risk of atrial fibrillation: a meta-analysis. *Journal of the American College of Cardiology*.

[132] Larsson SC, Drca N, Wolk A (2014). Alcohol consumption and risk of atrial fibrillation: a prospective study and dose-response meta-analysis. *Journal of the American College of Cardiology*.

[133] Djoussé L, Levy D, Benjamin EJ, Blease SJ, Russ A, Larson MG (2004). Long-term alcohol consumption and the risk of atrial fibrillation in the Framingham Study. *The American Journal of Cardiology*.

[134] Voskoboinik A, Kalman JM, De Silva A, Nicholls T, Costello B, Nanayakkara S (2020). Alcohol Abstinence in Drinkers with Atrial Fibrillation. *The New England Journal of Medicine*.

[135] Voskoboinik A, Wong G, Lee G, Nalliah C, Hawson J, Prabhu S (2019). Moderate alcohol consumption is associated with atrial electrical and structural changes: Insights from high-density left atrial electroanatomic mapping. *Heart Rhythm*.

[136] Mandyam MC, Vedantham V, Scheinman MM, Tseng ZH, Badhwar N, Lee BK (2012). Alcohol and vagal tone as triggers for paroxysmal atrial fibrillation. *The American Journal of Cardiology*.

[137] Takigawa M, Takahashi A, Kuwahara T, Takahashi Y, Okubo K, Nakashima E (2016). Impact of Alcohol Consumption on the Outcome of Catheter Ablation in Patients With Paroxysmal Atrial Fibrillation. *Journal of the American Heart Association*.

[138] Qiao Y, Shi R, Hou B, Wu L, Zheng L, Ding L (2015). Impact of Alcohol Consumption on Substrate Remodeling and Ablation Outcome of Paroxysmal Atrial Fibrillation. *Journal of the American Heart Association*.

[139] Huxley RR, Lopez FL, Folsom AR, Agarwal SK, Loehr LR, Soliman EZ (2011). Absolute and attributable risks of atrial fibrillation in relation to optimal and borderline risk factors: the Atherosclerosis Risk in Communities (ARIC) study. *Circulation*.

[140] Chamberlain AM, Agarwal SK, Folsom AR, Duval S, Soliman EZ, Ambrose M (2011). Smoking and incidence of atrial fibrillation: results from the Atherosclerosis Risk in Communities (ARIC) study. *Heart Rhythm*.

[141] Fukamizu S, Sakurada H, Takano M, Hojo R, Nakai M, Yuba T (2010). Effect of Cigarette Smoking on the Risk of Atrial Fibrillation Recurrence after Pulmonary Vein Isolation. *Journal of Arrhythmia*.

[142] Prineas RJ, Jacobs DR, Crow RS, Blackburn H (1980). Coffee, tea and VPB. *Journal of Chronic Diseases*.

[143] Cheng M, Hu Z, Lu X, Huang J, Gu D (2014). Caffeine intake and atrial fibrillation incidence: dose response meta-analysis of prospective cohort studies. *The Canadian Journal of Cardiology*.

[144] Krittanawong C, Tunhasiriwet A, Wang Z, Farrell AM, Chirapongsathorn S, Zhang H (2021). Is caffeine or coffee consumption a risk for new-onset atrial fibrillation? A systematic review and meta-analysis. *European Journal of Preventive Cardiology*.

[145] Bodar V, Chen J, Gaziano JM, Albert C, Djoussé L (2019). Coffee Consumption and Risk of Atrial Fibrillation in the Physicians’ Health Study. *Journal of the American Heart Association*.

[146] Chandan N, Matthews V, He H, Lachlan T, Lim VG, Joshi S (2024). Cardiac prehabilitation, rehabilitation and education in first-time atrial fibrillation (AF) ablation (CREED AF): Study protocol for a randomised controlled trial. *PloS One*.

[147] Hendriks JML, de Wit R, Crijns HJGM, Vrijhoef HJM, Prins MH, Pisters R (2012). Nurse-led care vs. usual care for patients with atrial fibrillation: results of a randomized trial of integrated chronic care vs. routine clinical care in ambulatory patients with atrial fibrillation. *European Heart Journal*.

[148] Wijtvliet EPJP, Tieleman RG, van Gelder IC, Pluymaekers NAHA, Rienstra M, Folkeringa RJ (2020). Nurse-led vs. usual-care for atrial fibrillation. *European Heart Journal*.

[149] van den Dries CJ, van Doorn S, Rutten FH, Oudega R, van de Leur SJCM, Elvan A (2020). Integrated management of atrial fibrillation in primary care: results of the ALL-IN cluster randomized trial. *European Heart Journal*.

[150] Yan H, Du YX, Wu FQ, Lu XY, Chen RM, Zhang Y (2022). Effects of nurse-led multidisciplinary team management on cardiovascular hospitalization and quality of life in patients with atrial fibrillation: Randomized controlled trial. *International Journal of Nursing Studies*.

